# The Effects of Berberine on the Gut Microbiota in Apc ^min/+^ Mice Fed with a High Fat Diet

**DOI:** 10.3390/molecules23092298

**Published:** 2018-09-08

**Authors:** Huan Wang, Lingnan Guan, Jing Li, Maode Lai, Xiaodong Wen

**Affiliations:** 1 State Key Laboratory of Natural Medicines, School of Basic Medicine and Clinical Pharmacy, China Pharmaceutical University, Nanjing 210009, China; wanghuanqc@126.com (H.W.); edo_1993@163.com (L.G.); lj_cpu@126.com (J.L.); lmd@cpu.edu.cn (M.L.); 2 Department of Pathology, School of Medicine, Zhejiang University, Hangzhou 310058, China

**Keywords:** berberine, Apc ^min/+^ mice, gut microbiota, high fat diet

## Abstract

**Background:** Berberine (BBR) has been extensively reported to inhibit colorectal cancer (CRC) development, though its bioavailability is poor. Nowadays, an increasing number of studies have shown that BBR significantly accumulates in the intestines and could regulate gut microbiota in obesity. The purpose of this study was to further explore the effects of BBR on gut microbiota in Apc ^min/+^ mice receiving a high fat diet (HFD). **Methods:** Apc ^min/+^ mice received either HFD alone or HFD and BBR for 12 weeks. The intestinal tissues were collected to evaluate the efficiency of BBR on neoplasm development by hematoxylin and eosin staining. Meanwhile, immunohistochemistry was conducted to investigate the effects of BBR on cyclin D1 and β-catenin in colon tissues. Fecal samples were subjected to 16S rRNA sequencing. **Results:** BBR significantly reduced intestinal tumor development and altered the structure of gut microbiota in Apc ^min/+^ mice fed with an HFD. At the phylum level, it was able to significantly inhibit the increase in *Verrucomicrobia*. At the genus level, it was able to suppress *Akkermansia* and elevate some short chain fat acid (SCFA)-producing bacteria. **Conclusions:** BBR significantly alleviated the development of CRC in Apc ^min/+^ mice fed with HFD and restored the enteric microbiome community.

## 1. Introduction

Colorectal cancer (CRC), which progresses from adenomas to adenocarcinomas, is a leading cause of cancer death worldwide [1]. It is widely accepted that this progression is triggered by the accumulation of both genetic and epigenetic factors [2]. Meanwhile, recently, and with increasing insight, people have realized that intestinal dysbiosis might be associated with CRC [3]. In adults, the bacterial population in the colon (10^11^–10^12^ CFU/g) is much larger compared with that in the stomach and small intestine (10^3^–10^4^ CFU/g). Accumulating evidence demonstrates that these bacteria are deeply involved in the prevention of CRC by multiple mechanisms. Firstly, normal diverse microbial communities can compete for attachment sites with pathogens, decreasing pathogen abundance and avoiding infections that induce carcinogenesis [4,5]. *Saccharomyces boulardii* could reduce the attachment of *Citrobacterrodentium* to epithelial cells by a reduction in the expression of EspB and Tir [6]. Secondly, microbial biofilms could grow on the mucosal surfaces of adenomas, leading to the disruption of mucous barriers and bacterial invasion into the epithelium [7]. In addition, the metabolites of gut microbiota have great effects on the progression of CRC. For example, dietary fiber could be fermented by bacteria in the colon to produce butyrate, which could suppress the growth of CRC by inhibiting histone deacetylase [8], while many Gram-negative bacteria, including *E. coli* and Helicobacter hepaticus, could produce cryptolectal distending toxins that induce cellular mutations, double-strand DNA breaks, and genomic instability [9]. Therefore, targeting of the gut microbiota might be a potential strategy for cancer interventions in the future.

As an isoquinoline alkaloid, berberine (BBR) is isolated from *Coptischinensis*, which is a famous traditional Chinese medicine [10]. It has been used for the treatment of intestinal infections for a long time in China and is one of the most popular herbal supplements for children in America [11]. Accumulating evidence has proven that BBR can suppress colon epithelial proliferation and tumorigenesis through the inhibition of epidermal growth factor receptor (EGFR) signaling, the phosphorylation of signal transducer and activator of transcription 3 (STAT3), and the activation of AMP-activated protein kinase (AMPK) [12,13,14]. However, we cannot fully explain its clinical efficacy, because its bioavailability is very low as a result of poor solubility and membrane permeability [15]. An increasing number of studies have shown that BBR significantly accumulates in the intestine [16] and could regulate gut microbiota in obesity [17,18] and steatohepatitis [19] animal models. Therefore, considering its low bioavailability and accumulation in the intestine, we hypothesized that BBR could regulate the gut microbiota, and at the same time, inhibit the development of CRC. In the current study, we evaluated the effect of BBR on the development of CRC in Apc ^min/+^ mice fed with a high fat diet (HFD). Furthermore, the effects of BBR on the modulation of gut microbiota in Apc ^min/+^ mice fed with a HFD were investigated by 16S rRNA sequencing.

## 2. Results

### 2.1. Effects of BBR on Intestinal Adenoma Development and β-Catenin Signaling in Apc ^min/+^ Mice Fed with a HFD

**Figure 1:**
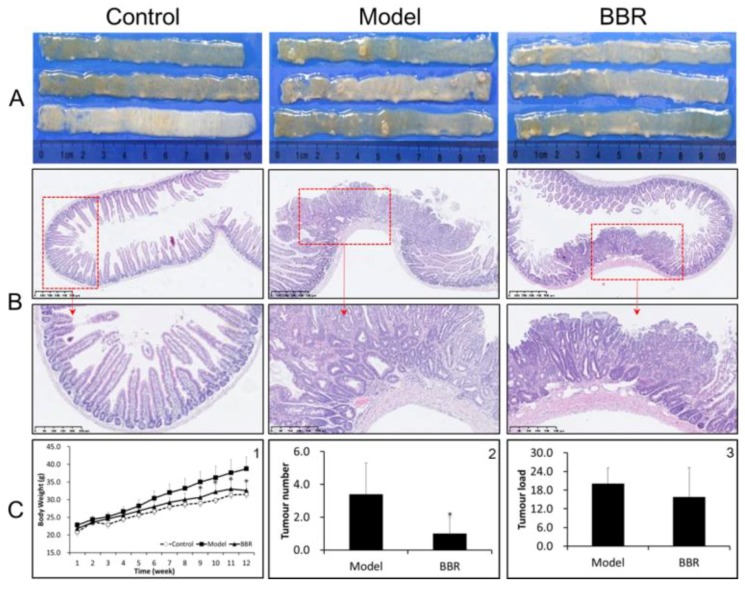
Berberine (BBR) attenuated intestinal adenoma progress in Apc ^min/+^ mice. (**A**) Representative macroscopic appearance of the small intestine (proximal) from wild type, Apc ^min/+^, and BBR treated Apc ^min/+^ mice. (**B**) Representative hematoxylin and eosin (HE) staining histological sections in the small intestine (proximal) in different groups: top panel: bar = 500 μm (100 μm × 5); bottom panel: bar = 250 μm (50 μm × 5). (**C-1**) Changes in body weight; (**C-2**) changes in tumor number; and (**C-3**) changes in tumor load in the small intestines of different groups. Control: wild type mice fed with a standard diet; model: Apc ^min/+^ mice fed with a high fat diet; BBR: Apc ^min/+^ mice fed with a high fat diet supplemented with BBR. The data are expressed as means ± standard errors. * represents *p* < 0.05 compared with the Model group.

**Figure 2:**
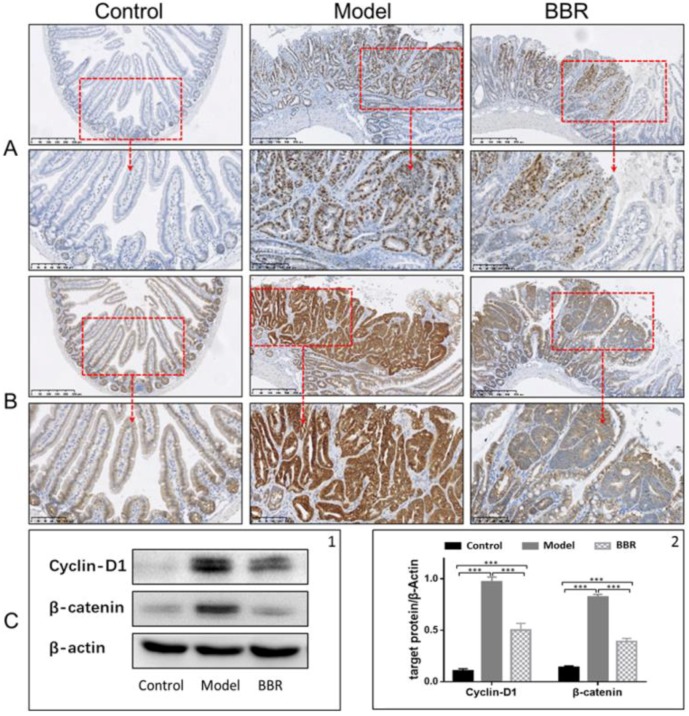
Effects of BBR on intestinal adenoma expression of β-catenin and cyclin D1 in Apc ^min/+^ mice. Immunostaining of cyclin D1 (**A**) and β-catenin (**B**) in different groups: top panel: bar = 250 μm (50 μm × 5); bottom panel: bar = 100 μm (20 μm × 5). (**C**) BBR downregulated the expression of cyclin D1 and β-catenin in the intestinal adenoma. (**C-1**) Representative results of three Western blotting repetitions. (**C-2**) The statistical results of three repetitions. Control: wild type mice fed with a standard diet; model: Apc ^min/+^ mice that received a high fat diet; BBR: Apc ^min/+^ mice that received a high fat diet and supplemented with BBR. The data are expressed as means ± standard errors. *** represents *p* < 0.001 compared with the indicated group.

**Figure 3:**
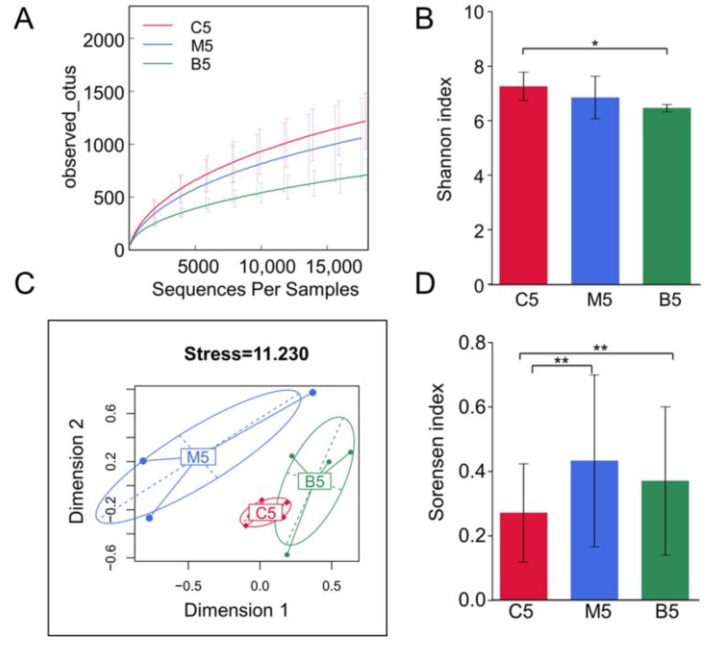
Diversity analysis of the gut microbiota among the three groups. (**A**) Rarefaction curves of the three groups basing on the observed operational taxonomic units (OTUs) obtained from different cutoff sequences. (**B**) Bar plots of alpha diversity distributions within the three groups using the Shannon index. (**C**) Multidimensional scaling (MDS) analysis of samples among the three groups using the Bray–Curtis dissimilarity matrices. (**D**) Bar plots of beta diversity distributions within the three groups using the Sorensen indices. C5: samples from wild type mice fed with a standard diet after 12 weeks; M5: samples from Apc ^min/+^ mice that received a high fat diet after 12 weeks; B5: samples from Apc ^min/+^ mice that received a high fat diet supplemented with BBR. Wilcoxon tests and Bonferroni corrections were used for the data analysis. * represents *p* < 0.05 and ** represents *p* < 0.01 compared with the indicated group.

In order to investigate the effect of BBR on tumor formation in Apc ^min/+^ mice, the mice were either treated or not treated with BBR for 12 weeks. The food intakes were similar in all groups. The bodyweight changes are shown in Figure 1C-1 and Figure S2. The weights of wild-type mice fed a standard diet (NCD) increased by approximately 10 g after the 12-week period, while the weights of the Apc ^min/+^ mice in the HFD group had higher increases of approximately 17 g. In contrast, BBR treatment obviously attenuated the increase in bodyweight. Meanwhile, after treatment with BBR, the tumor number and tumor load were significantly reduced (Figure 1A,C-2,C-3). A histological analysis of the tissues from the Apc ^min/+^ mice fed with an HFD showed that compared with normal crypts, their crypts were dysplasic, and their adenomas appeared with nuclear hyperchromasia and increased nucleus-to-cytoplasmic ratios (Figure 1B). However, in the BBR-treated group, these changes were greatly improved. Furthermore, immunohistochemistry and Western blot analysis showed that the expressions of cyclin D1 and β-catenin were upregulated in the Apc ^min/+^ mice fed with an HFD, while their expressions in BBR-treated mice were significantly reduced (Figure 2A–C). All of these results indicate that BBR could attenuate carcinogenesis in Apc ^min/+^ mice fed with an HFD. 

### 2.2. Overall Effects of BBR on the Gut Microbiota Structure in Apc ^min/+^ Mice Fed with an HFD

After submitting 15 fecal samples collected at week 12 to high throughput sequencing, we obtained 870,763 high quality sequences (with 29,025 ± 5199 sequences per sample), which were then delineated into 2456 operational taxonomic units (OTUs) at the 98% similarity threshold, as previously reported [17]. The refraction curves had already levelled off at this sequencing depth (Figure 3A), suggesting that these obtained sequences captured most of the sample information. As indicated by rarefaction curves and the Shannon index (Figure 3A,B), BBR administration decreased the alpha-diversity of the microbiota.

We collected samples in each group before treatment. Their gut microbiota were analyzed by principal component analysis (PCA). All the samples collected before treatment clustered together, indicating that the gut microbiota of the three groups were similar when we started our experiments (Figure S1). The overall structural changes of the gut microbiota after 12 weeks were analyzed by multidimensional scaling (MDS). An analysis using the Bray–Curtis dissimilarity matrices (Figure 3C) highlighted the overall differences in community compositions for the three groups. After 12 weeks of treatment, a clear separation was observed between Apc ^min/+^ mice fed with an HFD and the control group. Notably, the supplementary BBR restored the changes in the microbiota structure in Apc ^min/+^ mice fed with an HFD to almost to the level of the control group. Both the MDS plot and the Sorensen index (Figure 3D) showed that the within group variation of Apc ^min/+^ mice fed with an HFD was much bigger than that of the control group, while BBR treatment decreased the variation. All these results indicate that the intestinal microbial populations in Apc ^min/+^ mice fed with an HFD are significantly different from those in wild type mice, while BBR could restore these gut microbiota community changes.

### 2.3. Effects of BBR on Gut Microbiota in Apc ^min/+^ Mice Fed with an HFD at Different Levels

**Figure 4:**
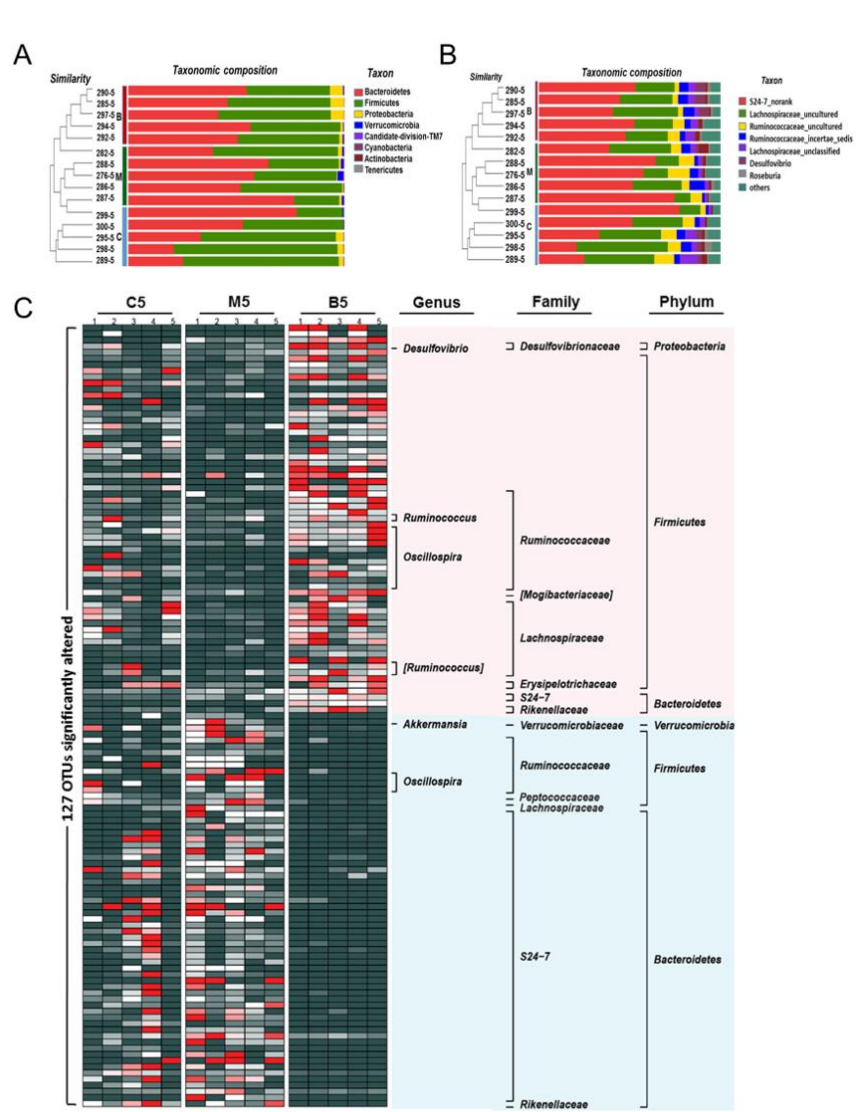
Effects of BBR on the gut microbiota compositions among the three groups. (**A**) Bar plot comparing the phyla compositions of different groups superposed on the clustering tree. (**B**) Bar plot comparing the genera compositions of different groups superposed on the clustering tree. (**C**) Heatmap visualizing the relative abundance of the detected top 127 predictive operational taxonomic units (OTUs). The red color represents high abundance, while the dark color represents low abundance. The labels on the right represent the taxonomy of each OTU. C5: samples from wild type mice fed with a standard diet after 12 weeks; M5: samples from Apc ^min/+^ mice fed with a high fat diet after 12 weeks; B5: samples from Apc ^min/+^ mice fed with a high fat diet supplemented with BBR after 12 weeks.

The taxonomic results at the phylum level (Figure 4A and Table S1) revealed that *Firmicutes* and *Bacteroidetes* were the most predominant phyla in the gut microbiota of wild type mice, accounting for 55.7% and 42.28%, respectively. Meanwhile, *Proteobacteria* and *Verrucomicrobia* constituted the second most abundant phyla, contributing 1.69% and 0.11% in the wild type mice. *Bacteroidetes* accounted for 21.2–77.97%, and *Firmicutes* varied from 21.2–75.8% in all the wild type mice, suggesting that high inter-individual variability existed in the microbial composition among samples. However, a significant increase in the level of *Verrucomicrobia* (*p* < 0.05) was still observed between wild type and Apc ^min/+^ mice fed with an HFD. In contrast, after BBR treatment, the levels of *Verrucomicrobia* decreased from 1.12% to 0.2% (Figure 4B and Table S1). Although the levels of *Firmicutes* and *Bacteroidetes* showed no significant differences between groups, there was still a trend for BBR treatment to decrease the level of *Bacteroidetes* while upregulating the level of *Firmicutes,* compared with levels in the Apc ^min/+^ mice fed with an HFD. These results suggest that BBR could regulate the gut microbiota, especially the *Verrucomicrobia*.

**Figure 5:**
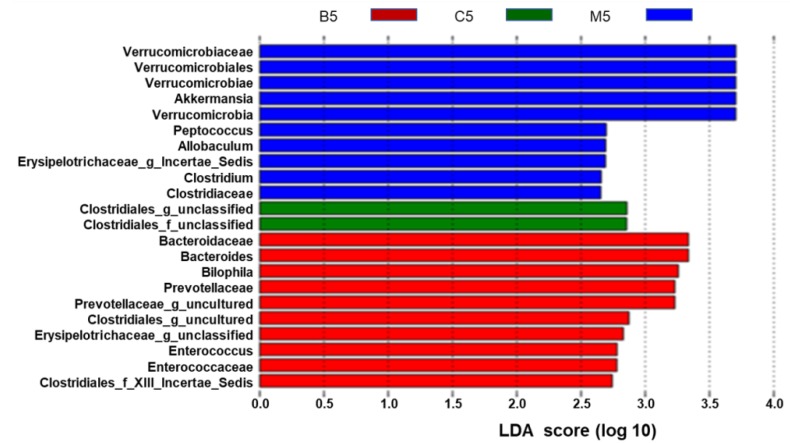
Different structures of gut microbiota among the three groups. The ten blue bars represent the ten microbial communities in the model group that are significant different from those in the control and BBR groups. The two green bars represent the two microbial communities in the control group that are significant different from those in the model and BBR groups. The ten red bars represent ten microbial communities in the BBR group that are significantly different from those in the control and model groups—the higher the linear discriminant analysis (LDA) score, the more important the role of the microbiota in a particular group.

At the genus level, the ten most abundant genera in wild type mice, accounting for approximately 96% of all assigned sequence readings (Figure 4B and Table S2), were *S24-7_norank* (41.63%), *Lachnospiraceae*_uncultured (32.37%), *Ruminococcaceae*_uncultured (7.28%), *Ruminococcaceae*_*incertae_sedis* (4.60%), *Lachnospiraceae*_unclassified (3.65%), *Desulfovibrio* (1.68%), *Roseburia* (1.47%), *Ruminococ-caceae*_unclassified (1.54%), *Anaerotruncus* (1.02%), and *Oscillibacter* (1.12%). The types of genera did not change too much between the HFD group and the BBR group (Figure 4A and Table S2), while the levels of each genus statistically changed (Figure 4B and Table S2). In the HFD group, *Akkermansia*, *Bacteroides*, and *Prevotellaceae*_uncultured were enriched, while *Lachnospiraceae*_*incertae*_ *sedis* was inhibited. In contrast, these phenomena were reversed in the BBR group, indicating that BBR could modulate the abundance of *Akkermansia* and *Lachnospiraceae*_*incertae*_*sedis*. Furthermore, the cladogram obtained from the LEfSe analysis and the linear discriminant analysis (LDA) score analysis showed the predominant bacteria of the microbiota in the three groups (Figure 5 and Figure S3). *Verrucomicrobia*, *Akkermansia*, and *Verrucomicrobiae* were enriched in the HFD group, whereas the predominant communities of the BBR group were *Bacteroidaceae*, *Bacteroides*, and *Bilophila*. 

## 3. Discussion

A variety of factors contribute to the etiology of CRC, including genetic mutations, diet, and lifestyle. At the same time, previous studies have demonstrated that gut bacteria also play a key role in the pathogenesis of CRC. It has been reported that the levels of *Fusobacterium nucleatum* and *Clostridium difficile* dramatically increase in the intestinal microbiota of CRC patients [20], demonstrating that the composition of the gut microbiota in CRC patients might be altered. Therefore, developing agents that could targeted at gut microbiota could be a new way to combat cancer.

BBR has been used as an anti-infection agent for thousands of years. It has been proven that BBR could induce cell apoptosis in colon cancer cells through multiple signalling pathways [21] and could potently attenuate intestinal polyp growth in animal models [22]. Meanwhile, BBR could improve microbiota structures, thereby contributing to its anti-obesity and anti-diabetes abilities [17]. However, whether or not BBR can regulate the gut microbiota during the CRC process was still previously unknown. In this study, we used Apc ^min/+^ mice, an Apc gene mutation animal model, to evaluate the effect of BBR on CRC. Meanwhile, numerous epidemiologic and clinical studies have linked the obese state to an increased colon cancer risk and/or mortality [23]. Although Apc ^min/+^ mice is a tumorigenic phenotype that can develop intestinal tumors, dietary modification with high fat could accelerate the carcinogenesis process just as high fat diets promote CRC incidence in humans, making this animal model more capable of reflecting the CRC process in humans [24]. Thus, we used Apc ^min/+^ mice fed with an HFD to reflect the gene–diet interactions in carcinogenesis. Using comparisons between the model group and BBR group, we investigated whether or not BBR could change the gut microbiota in the model group. However, we do not know whether these changes are good or bad for carcinogenesis. The gut microbiota in the control group represent the normal condition. From comparisons among the control, model, and BBR groups, we found that supplementation with BBR restored the changes in the microbiota structure in the model group so that the structure was almost the same as in the control group, indicating that its ability to regulate the enteric microbiome community might be beneficial for ameliorating carcinogenesis (Figure S4). In the present study, we only focused on whether BBR could regulate the microbiota structure in Apc ^min/+^ mice fed an HFD, a colonic tumorigenesis model integrated with genetic mutations and environmental factors. In future studies, we will add a wild type mice/HFD group and the Apc ^min/+^ mice/NCD group to investigate the effects of BBR induced by genetic mutations or HFD on the microbiome community in detail. 

In the present study, we proved that BBR significantly ameliorates CRC progression in Apc ^min/+^ mice fed with an HFD, as evidenced by the reduced numbers of tumors and decreased inflammation in colon tissues. These results are consistent with previous studies, which have shown that BBR could potently attenuate the growth of intestinal polyps in Apc ^min/+^ mice [22]. The abnormal activation of Wnt/β–catenin, the signalling pathway, was shown to be an important event in CRC development. In Apc ^min/+^ mice, because of the Apc gene mutation, β-catenin degradation is blocked, leading to the nuclear translocation of β-catenin, which promotes the transcription of target genes [25]. We observed β-catenin accumulation in Apc ^min/+^ mice, and BBR treatment could suppress this process, supporting the therapeutic potential of BBR on CRC.

Bacteria are one of the key constituents of the human body. In the present study, the results of multivariate statistics indicated that imbalances in the microbiota occurred in Apc ^min/+^ mice fed with an HFD, while BBR treatment alleviated the imbalances. Notably, the number of *Verrucomicrobia* was significantly enriched in Apc ^min/+^ mice fed with an HFD, while it was down-regulated after BBR treatment. It has been reported that *Verrucomicrobia* exhibit proinflammatory properties and their level of accumulation is increased in AOM/DSS mice and DSS mice [7,26]. Meanwhile, previous studies have also shown that *Bacteroidetes* exhibit proinflammatory properties in several animal models of IBD through the production of mucin degrading sulfatases and destroy the intestinal mucosal barrier [27]. In our study, because of inter-individual variability, the level of *Bacteroidetes* did not show significant differences between groups. However, there was still a trend for the abundance of *Bacteroidetes* to be upregulated in the HFD group and decreased after BBR treatment. Consistent with the above results, at the genus level, *Akkermansia*, which belongs to *Verrucomicrobia*, was significantly increased in the HFD group. It has been shown in previous reports that the abundance of *Akkermansia* is strongly linked to the rate of tumorigenesis. The abundance of *Akkermansia* was enhanced in CRC patients [28]. It is also known to be a mucin degrader that is associated with intestinal inflammation and is positively correlated with tumor incidence [29]. BBR treatment could decrease the abundance of *Akkermansia*, suggesting that the blocking of mucin degradation might be one of the mechanisms behind the therapeutic effect of BBR.

One of the benefits of the gut microbiome is that it can ferment carbohydrates (e.g., fiber) into short-chain fatty acids (SCFA), such as acetic acid and butyrate. Butyrate could alleviate inflammation and improve intestinal epithelial barrier function, thereby inhibiting the growth of cancer cells [30]. The loss of butyrate-producing bacteria has been frequently observed in patients with CRC and metabolic diseases [17]. It has been reported that the abundance of *Roseburia* and *Eubacterium*, two butyrate-producing bacteria, is reduced in CRC rats [31], and the abundance of *Lachnospiraceae* is also reduced in CRC patients [31]. In our study, the abundance of *Lachnospiraceae*, the butyrate-producing bacteria, decreased in Apc ^min/+^ mice fed with an HFD. However, this imbalance was reverted by BBR treatment, suggesting that BBR could upregulate some butyrate-producing bacteria in the intestines. This result is consistent with previous studies that have shown that BBR can selectively modulate SCFA-producing bacteria in HFD-induced obese rats [17]. However, the measurement of intestinal SCFA concentrations is still needed to provide a better understanding of the mechanism through which BBR acts on SCFA-producing bacteria.

## 4. Materials and Methods 

### 4.1. Chemicals and Reagents

Berberine chloride (with a purity of 98.0%) was bought from Chengdu Must Biological Technology Co., Ltd. (Chengdu, China). A QIAamp DNA stool minikit was obtained from Qiagen China Co., Ltd. (Shanghai, China). Both the Western (20% fat) and control normal diets were obtained from Jiangsu Synergic Bioengineering Company (Nanjing, China). The anti-β-catenin (#ab32572) and anti-cyclin D1 (#ab134175) antibodies were purchased from Abcam (Abcam, Shanghai, China) for IHC and Western blotting analysis. The secondary antibodies were sheep anti-mouse IgG (A0129) and sheep anti-rabbit IgG (A0208, Beyotime Institute of Biotechnology, Shanghai, China).

### 4.2. Animals and Experimental Design

All the experiments were conducted in accordance with these protocols and were approved by the Institutional Animal Care and Use Committee of China Pharmaceutical University (Approval date: 5 February 2015; No. 201400175). Ten male C57BL/6J-Apc ^min/+^ mice and five male wild-type C57BL/6 were obtained from the Model Animal Research Centre of Nanjing University. Mice were housed in sterilized cages in a room in which the temperature was held constant at 23 ± 2 °C, with the humidity controlled at 55% ± 5% on a standard light/dark cycle. All the mice were free to consume food and water throughout the experimental period. Berberine was blended into the diet with a mixer. The Apc ^min/+^ mice were randomized into the HFD group (received HFD) and berberine group (received HFD supplemented with 500 ppm of berberine) at six weeks of age. The wild-type C57BL/6 mice consumed the NCD diet and were used as the control. Their faeces samples were collected before treatment. After 12 weeks of treatment, mice were sacrificed, and the gut samples of all animals were collected and kept at −80 °C or in 10% buffered formalin. The faeces samples were also collected and stored at −80 °C prior to the 16S rRNA gene profiling analysis. 

### 4.3. Histological Examination

All the gut samples were washed with ice-cold PBS, opened longitudinally, and cut into four segments (I–III from the proximal (I) to distal small intestine (III), and colon). Images of the intestines were obtained via high-definition digital cameras. The number of intestinal polyps was counted, and their sizes were measured with image processing and analysis software. The gut tissue samples were embedded in paraffin and then were sectioned and stained with hematoxylin and eosin (H&E). r 2.0 HT was used for the scanning of samples.

### 4.4. Immunohistochemistry

The paraffin embedded samples were cut into flip-flops (5 µM). The histological characterizations were carried out as previously described [32].

### 4.5. Western Blotting Analysis

The total protein of intestinal tissues was extracted by grinding in RIPA lysis buffer. The Western blot procedure was carried out as previously described [14].

### 4.6. DNA Extraction, PCR Amplification, and Illumina MiSeq Sequencing

The extraction of total genomic DNA from faeces samples was conducted with the QIAamp DNA stool Mini Kit (Qiagen, Shanghai, China). The bacterial 16S ribosomal RNA genes in the V4–V5 region were amplified by bar-coded primers (338F 5’-barcode-ACTCCTACGGGAGGCAGCAG-3’ and 806R 5’-GGACTACHVGGGTWTCTAAT-3’), with a touchdown PCR procedure. The purification of amplicons was carried out with a DNA Gel Extraction Kit from Axygen Biosciences (Union City, CA, USA). After purification, amplicons were combined and submitted to sequencing on an Illumina MiSeq platform using standard protocols. The criteria and procedures used for the processing of sequencing data were the same as previously reported [33].

### 4.7. Statistical Analysis

All data are expressed as means ± SDs. Data were analysed by *t*-tests and Mann–Whitney tests in SPSS statistics (IBM SPSS, Chicago, IL, USA) (version 22.0). *p* < 0.05 was considered statistically significant.

## 5. Conclusions

We have confirmed that BBR has anti-CRC effects and could restore the enteric microbiome community to a healthy state. It suppressed *Akkermansia* and elevated some SCFA-producing bacteria (e.g., *Lachnospiraceae*) in Apc ^min/+^ mice that received an HFD. Our research suggests that BBR may prevent CRC and restore the enteric microbiome community in Apc ^min/+^ mice receiving an HFD.

## Supplementary Materials

Supplementary materials are available online. Table S1: Significantly different genus between groups as revealed by taxon-based comparisons.; Figure S1: PCA score plot for both 0-week collected and 12-week collected samples.; Figure S2: Food intake per mouse per day in the experimental period.; Figure S3: Taxonomic representation of statistically and biologically consistent differences in three groups.; Figure S4: Animal groups in this study.

## References

[1] Liu H., Huang C., Wu L., Wen B. (2016). Effect of evodiamine and berberine on miR-429 as an oncogene in human colorectal cancer. Onco Targets Ther..

[2] Lam K., Pan K., Linnekamp J.F., Medema J.P., Kandimalla R. (2016). DNA methylation based biomarkers in colorectal cancer: A systematic review. Biochim. Biophys. Acta.

[3] Wang X., Huycke M.M. (2015). Colorectal cancer: Role of commensal bacteria and bystander effects. Gut Microbes.

[4] Bultman S.J. (2016). The microbiome and its potential as a cancer preventive intervention. Semin. Oncol..

[5] Kang Y., Pan W., Cai Y. (2017). Gut microbiota and colorectal cancer: Insights into pathogenesis for novel therapeutic strategies. Gastroenterol..

[6] Wu X., Vallance B.A., Boyer L., Bergstrom K.S., Walker J., Madsen K., O’Kusky J.R., Buchan A.M., Jacobson K. (2008). Saccharomyces boulardii ameliorates Citrobacterrodentium-induced colitis through actions on bacterial virulence factors. Am. J. Physiol. Gastrointest. Liver Physiol..

[7] Wang C.Z., Yu C., Wen X.D., Chen L., Zhang C.F., Calway T., Qiu Y., Wang Y., Zhang Z., Anderson S. (2016). American Ginseng Attenuates Colitis-Associated Colon Carcinogenesis in Mice: Impact on Gut Microbiota and Metabolomics. Cancer Prev. Res. (Phila).

[8] Hu S., Liu L., Chang E.B., Wang J.Y., Raufman J.P. (2015). Butyrate inhibits pro-proliferative miR-92a by diminishing c-Myc-induced miR-17-92a cluster transcription in human colon cancer cells. Mol. Cancer.

[9] Wang X., Yang Y., Huycke M.M. (2017). Microbiome-driven carcinogenesis in colorectal cancer: Models and mechanisms. Free Radical. Biol. Med..

[10] Zhang X., Zhao Y., Zhang M., Pang X., Xu J., Kang C., Li M., Zhang C., Zhang Z., Zhang Y. (2012). Structural changes of gut microbiota during berberine-mediated prevention of obesity and insulin resistance in high-fat diet-fed rats. PLoS ONE.

[11] Guo Y., Zhang Y., Huang W., Selwyn F.P., Klaassen C.D. (2016). Dose-response effect of berberine on bile acid profile and gut microbiota in mice. BMC Complement. Altern. Med..

[12] Habtemariam S. (2016). Berberine and inflammatory bowel disease: A concise review. Pharmacol. Res..

[13] Li D., Zhang Y., Liu K., Zhao Y., Xu B., Xu L., Tan L., Tian Y., Li C., Zhang W. (2017). Berberine inhibits colitis-associated tumorigenesis via suppressing inflammatory responses and the consequent EGFR signaling-involved tumor cell growth. Lab. Investig..

[14] Li W., Hua B., Saud S.M., Lin H., Hou W., Matter M.S., Jia L., Colburn N.H., Young M.R. (2015). Berberine regulates AMP-activated protein kinase signaling pathways and inhibits colon tumorigenesis in mice. Mol. Carcinog..

[15] Zhao J.M., Ming Z., Shen G.W., Xiao J.B., Hatch G.M., Jing K.G., Li C. (2014). Amorphous solid dispersion of berberine with absorption enhancer demonstrates a remarkable hypoglycemic effect via improving its bioavailability. Int. J. Pharm..

[16] Sun R., Yang N., Kong B., Cao B., Feng D., Yu X., Ge C., Huang J., Shen J., Wang P. (2017). Orally Administered Berberine Modulates Hepatic Lipid Metabolism by Altering Microbial Bile Acid Metabolism and the Intestinal FXR Signaling Pathway. Mol. Pharmacol..

[17] Zhang X., Zhao Y., Xu J., Xue Z., Zhang M., Pang X., Zhang X., Zhao L. (2015). Modulation of gut microbiota by berberine and metformin during the treatment of high-fat diet-induced obesity in rats. Sci. Rep..

[18] Xu J.H., Liu X.Z., Pan W., Zou D.J. (2017). Berberine protects against diet-induced obesity through regulating metabolic endotoxemia and gut hormone levels. Mol. Med. Rep..

[19] Cao Y., Pan Q., Cai W., Shen F., Chen G.Y., Xu L.M., Fan J.G. (2016). Modulation of Gut Microbiota by Berberine Improves Steatohepatitis in High-Fat Diet-Fed BALB/C. Mice. Arch. Iran. Med..

[20] Fukugaiti M.H., Ignacio A., Fernandes M.R., Ribeiro J.U., Nakano V., Avila-Campos M.J. (2015). High occurrence of Fusobacterium nucleatum and Clostridium difficile in the intestinal microbiota of colorectal carcinoma patients. Braz. J. Microbiol..

[21] Chidambara M.K.N., Jayaprakasha G.K., Patil B.S. (2012). The natural alkaloid berberine targets multiple pathways to induce cell death in cultured human colon cancer cells. Eur. J. Pharmacol..

[22] Zhang J., Cao H., Zhang B., Cao H., Xu X., Ruan H., Yi T., Tan L., Qu R., Song G. (2013). Berberine potently attenuates intestinal polyps growth in ApcMin mice and familial adenomatous polyposis patients through inhibition of Wnt signalling. J. Cell Mol. Med..

[23] Calle E.E., Thun M.J. (2004). Obesity and cancer. Oncogene.

[24] Yang K., Edelmann W., Fan K., Lau K., Leung D., Newmark H., Kucherlapati R., Lipkin M. (1998). Dietary modulation of carcinoma development in a mouse model for human familial adenomatous polyposis. Cancer Res..

[25] Talla S.B., Brembeck F.H. (2016). The role of Pygo2 for Wnt/β-catenin signaling activity during intestinal tumor initiation and progression. Oncotarget.

[26] Nagalingam N.A., Kao J.Y., Young V.B. (2011). Microbial ecology of the murine gut associated with the development of dextran sodium sulfate-induced colitis. Inflamm. Bowel Dis..

[27] Lucke K., Miehlke S., Jacobs E., Schuppler M. (2006). Prevalence of Bacteroides and Prevotella spp. in ulcerative colitis. J. Med. Microbiol..

[28] Wu M., Wu Y., Deng B., Li J., Cao H., Qu Y., Qian X., Zhong G. (2016). Isoliquiritigenin decreases the incidence of colitis-associated colorectal cancer by modulating the intestinal microbiota. Oncotarget.

[29] Baxter N.T., Zackular J.P., Chen G.Y., Schloss P.D. (2014). Structure of the gut microbiome following colonization with human feces determines colonic tumor burden. Microbiome.

[30] Zackular J.P., Rogers M.A., Ruffin M.T. (2014). The human gut microbiome as a screening tool for colorectal cancer. Cancer Prev. Res. (Phila).

[31] Zhu Q., Jin Z., Wu W., Gao R., Guo B., Gao Z., Yang Y., Qin H. (2014). Analysis of the intestinal lumen microbiota in an animal model of colorectal cancer. PLoS ONE.

[32] Wen X.D., Wang C.Z., Yu C., Zhao L., Zhang Z., Matin A., Wang Y., Li P., Xiao S.Y., Du W. (2014). Panax noto ginseng attenuates experimental colitis in the azoxymethane/dextran sulfate sodium mouse model. Phytother. Res..

[33] Song X., Xu Z.M., Liu Q., Li Y., Ma Y., Wang J., Sun M.R., Shao H.B., Sun H., Malin G. (2016). Comparative study of the composition and genetic diversity of the picoeukaryote community in a Chinese aquaculture area and an open sea area. J. Mar. Biol. Assoc. UK.

