# Supplementary material for: The Effects of Berberine on the Gut Microbiota in Apc min/+ Mice Fed with a High Fat Diet
Source: Molecules. 2018 Sep 8;23(9):2298. doi: 10.3390/molecules23092298 (PMC6225274; doi:10.3390/molecules23092298)
Supplement: Supplementary file 1 [file molecules-23-02298-s001.zip › Supplementary Materials.pdf]

Table S1. Significantly different genus between groups as revealed by taxon-based comparisons

Taxon	C5 %	B5 %	M5 %	C5 vs M5 p value	B5 vs M5 p value
S24-7_norank	41.63	48.02	57.5	0.175	0.251
Lachnospiraceae_uncultured	32.37	25.97	19.14	0.117	0.251
Ruminococcaceae_uncultured	7.28	4.29	7.41	0.754	0.117
***Ruminococcaceae_incertae_sedis**	**4.6**	**3.72**	**2.1**	**0.175**	**0.016**
Lachnospiraceae_unclassified	3.65	4.61	4.35	0.917	0.602
***Desulfovibrio**	**1.68**	**3.8**	**0.77**	**0.602**	**0.028**
Roseburia	1.47	1.38	0.89	0.917	0.116
Ruminococcaceae_unclassified	1.54	1.47	2	0.917	0.917
Anaerotruncus	1.02	1.07	0.97	0.754	0.602
**#*Akkermansia**	**0.11**	**0.2**	**1.12**	**0.028**	**0.047**
Oscillibacter	1.12	0.82	0.87	0.602	0.834
***Alistipes**	**0.33**	**1.02**	**0.29**	**0.402**	**0.047**
**#*Lachnospiraceae_incertae_sedis**	**0.5**	**0.42**	**0**	**0.005**	**0.005**
Ruminococcus	0.92	0.39	0.32	0.175	0.53
Odoribacter	0.32	0.35	0.15	0.249	0.175
**#Bacteroides**	**0.00**	**0.44**	**0.18**	**0.008**	**0.173**
**#Prevotellaceae_uncultured**	**0.00**	**0.34**	**0.15**	**0.007**	**0.834**
Escherichia-Shigella	0	0	0.08	0.419	0.572

# $p<0.05$: Control vs.Model; * $p<0.05$: BBR vs.Model

Table S2. Significantly different phylum between groups as revealed by taxon-based comparisons

Taxon	C5(%)	B5(%)	M5(%)	C5 vs M5 p value	B5 vs M5 p value
Bacteroidetes	42.28	50.17	58.27	0.251	0.251
Firmicutes	55.7	45.37	39.48	0.251	0.465
***Proteobacteria**	**1.69**	**4.17**	**1.01**	**0.602**	**0.028**
**#*Verrucomicrobia**	**0.11**	**0.2**	**1.12**	**0.028**	**0.047**

# $p<0.05$: Control vs.Model; * $p<0.05$: BBR vs.Model

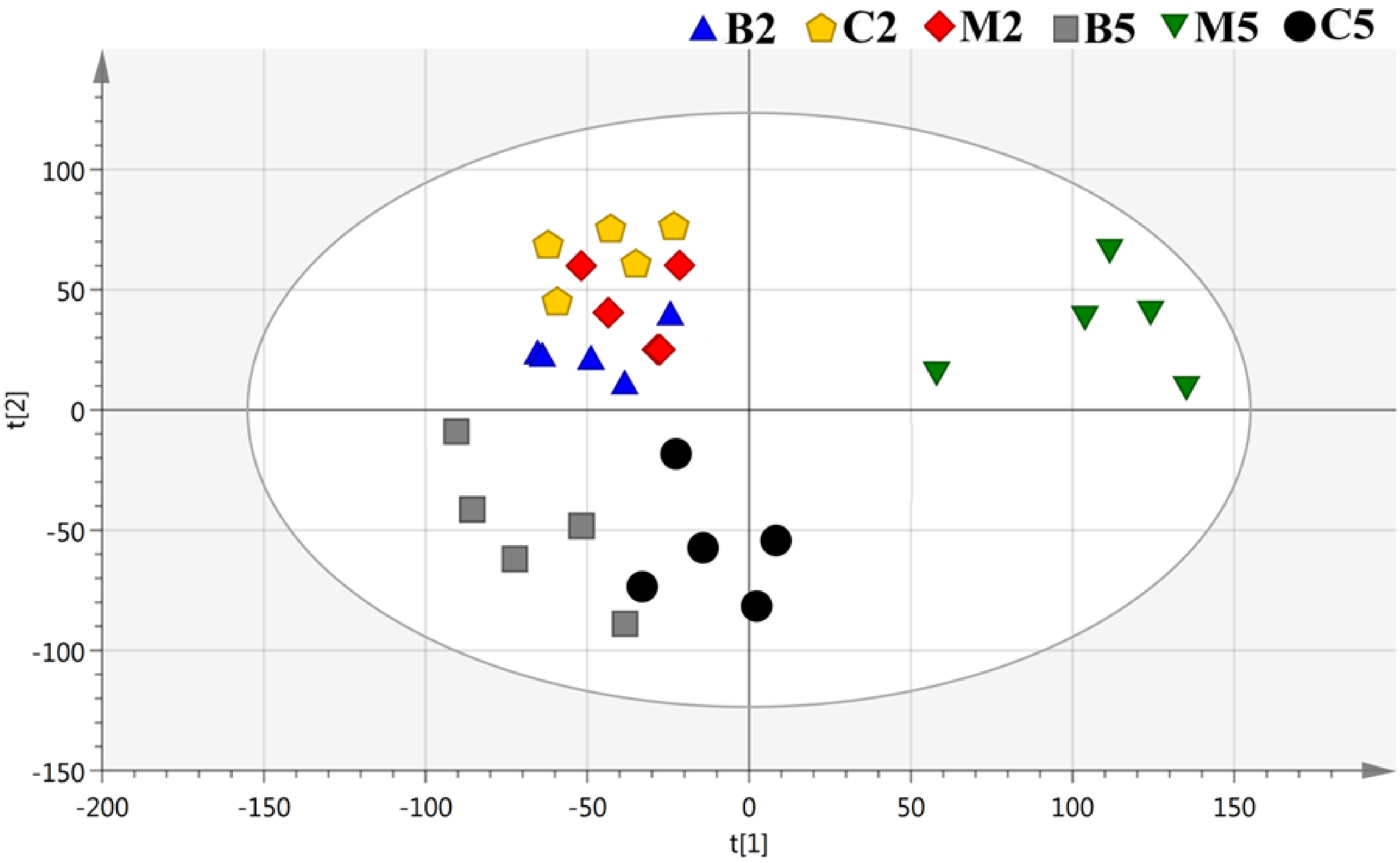

Figure S1. PCA score plot for both 0-week collected and 12-week collected samples. C5: samples from wild type mice fed with standard diet after 12 weeks; M5: samples from Apc $^{min/+}$ mice fed with high fat diet after 12 weeks; B5: samples from Apc $^{min/+}$ mice fed with high fat diet and BBR supplemented after 12 weeks. C2: samples from wild type mice fed with standard diet after 0 weeks; M2: samples from Apc $^{min/+}$ mice fed with high fat diet after 0 weeks; B2: samples from Apc $^{min/+}$ mice fed with high fat diet and BBR supplemented after 0 weeks.

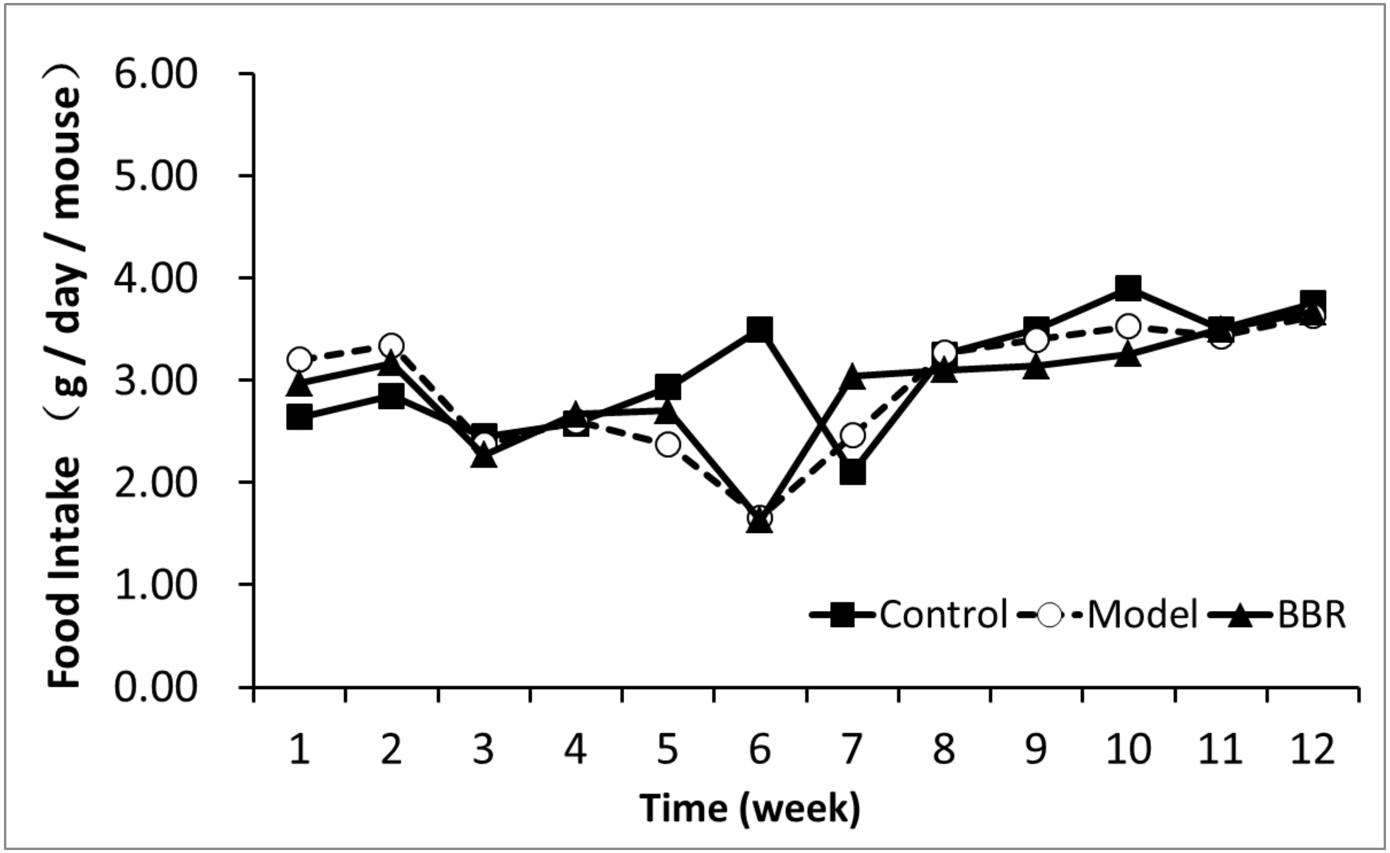

Figure S2. Food intake per mouse per day in the experimental period.

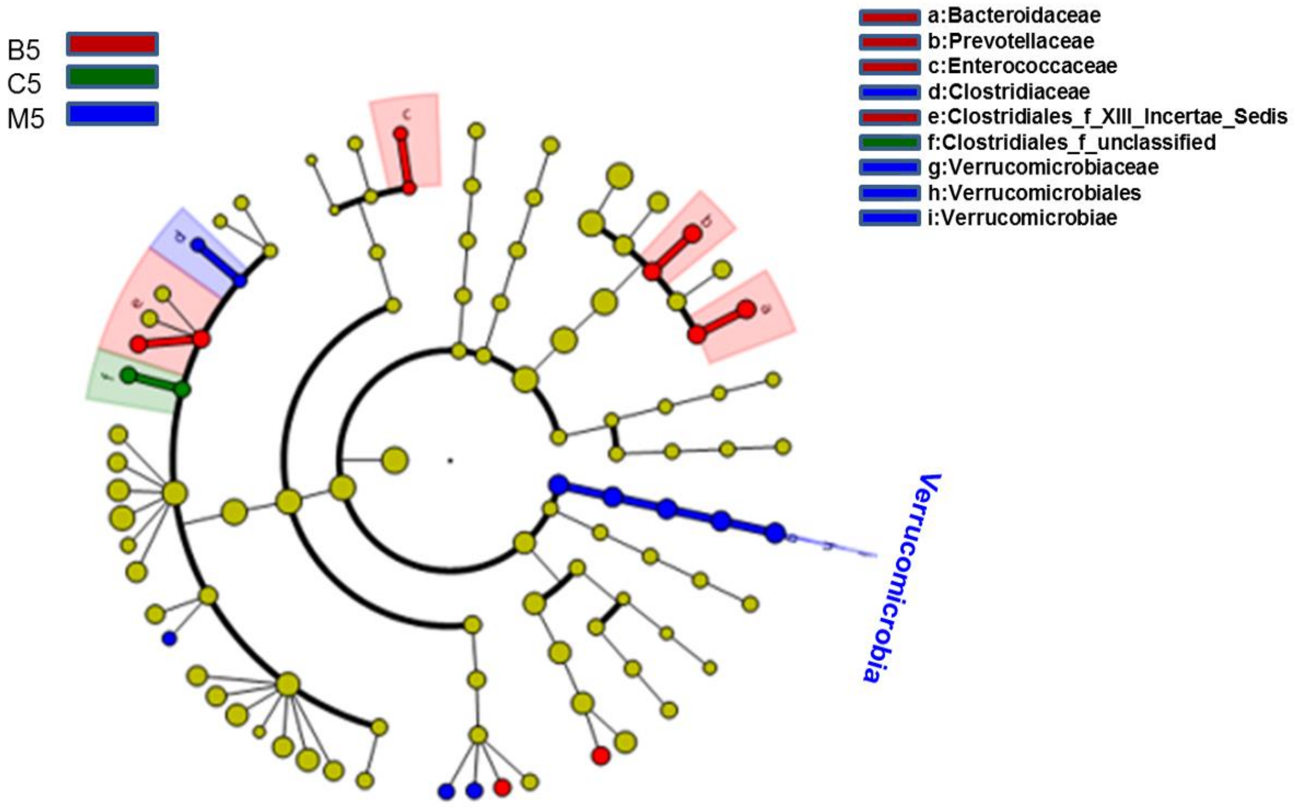

Figure S3. Taxonomic representation of statistically and biologically consistent differences in three groups.

Differences are represented by the color of the most abundant class (red indicating berberine group, green for control group, blue indicating model group and yellow for non-significant). The diameter of each circle is proportional to the taxon’s abundance.

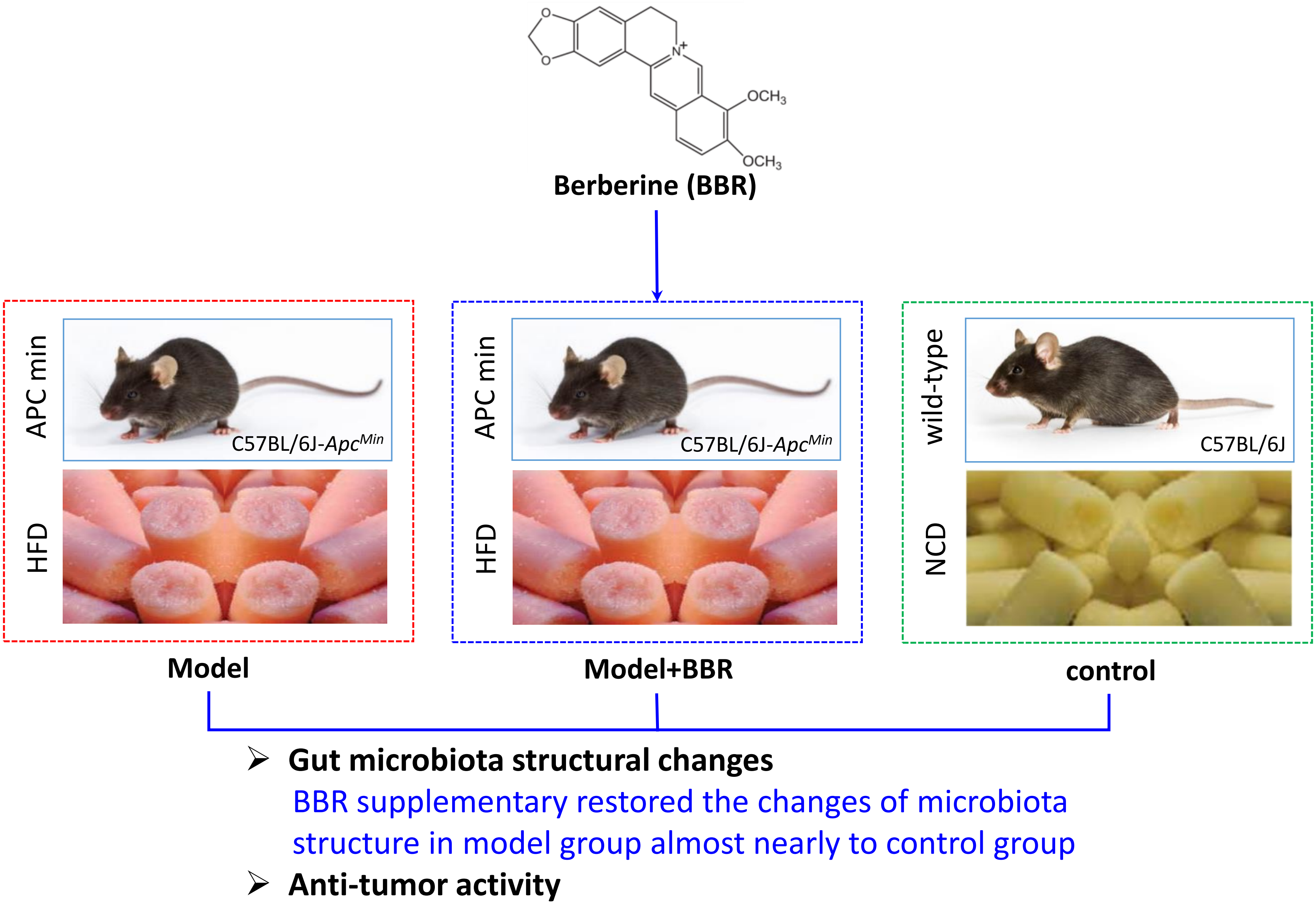

Figure S4 Animal groups in this study
